# Yeast Extract: Characteristics, Production, Applications and Future Perspectives

**DOI:** 10.4014/jmb.2207.07057

**Published:** 2022-10-17

**Authors:** Zekun Tao, Haibo Yuan, Meng Liu, Qian Liu, Siyi Zhang, Hongling Liu, Yi Jiang, Di Huang, Tengfei Wang

**Affiliations:** 1 State Key Laboratory of Bio-Based Material and Green Papermaking (LBMP), Qilu University of Technology (Shandong Academy of Sciences), Jinan 250353, Shandong, P.R. China; 2 Key Laboratory of Shandong Microbial Engineering, School of Bioengineering, Qilu University of Technology (Shandong Academy of Sciences), Jinan 250353, Shandong, P.R. China

**Keywords:** Waste yeast, yeast extract, nitrogen source, polysaccharides, food additives

## Abstract

Yeast extract is a product prepared mainly from waste brewer’s yeast, which is rich in nucleotides, proteins, amino acids, sugars and a variety of trace elements, and has the advantages of low production cost and abundant supply of raw material. Consequently, yeast extracts are widely used in various fields as animal feed additives, food flavoring agents and additives, cosmetic supplements, and microbial fermentation media; however, their full potential has not yet been realized. To improve understanding of current research knowledge, this review summarizes the ingredients, production technology, and applications of yeast extracts, and discusses the relationship between their properties and applications. Developmental trends and future prospects of yeast extract are also previewed, with the aim of providing a theoretical basis for the development and expansion of future applications.

## Introduction

As living standards in most countries have improved, consumer demand for healthy, nutritious and safe food has steadily increased. Yeast extract, which is safe and nutritious, is now considered a natural, high-quality product capable of meeting diverse food flavor requirements and supplying essential dietary nutrients [1, 2]. Yeast extract is usually defined as the water-soluble extract produced from yeast waste streams (*e.g.*, baker's yeast, brewer's yeast, *Candida utilis*, *Candida tropicalis*, and *Kluyveromyces marxianus*) [3] following disruption of the cell membrane by various means [4]. Also known as “yeast hydrolysate” in the food industry, yeast extract is regarded as Generally Recognized as Safe (GRAS) by most food safety certification bodies around the world [5]. In particular, yeast extract has attracted increasing attention because of its low production cost, wide range of sources, and high content of vitamins, proteins, and minerals needed by the food industry. For example, Elsa *et al*.[6] obtained yeast extract by mechanical crushing and found the protein content to be as high as 64.1% (dw), the fat content at only 1.32% (dw), and the RNA content at 4%. Meanwhile, the essential amino acids accounted for 40% of total amino acid, while those with flavor-enhancing functions (glutamic acid, aspartic acid, glycine, and alanine) accounted for 34% of total amino acid. In addition to the great variety of physiologically valuable substances in yeast extract, its high antioxidant capacity is also very useful to the food industry [4].

**Fig. 1:**
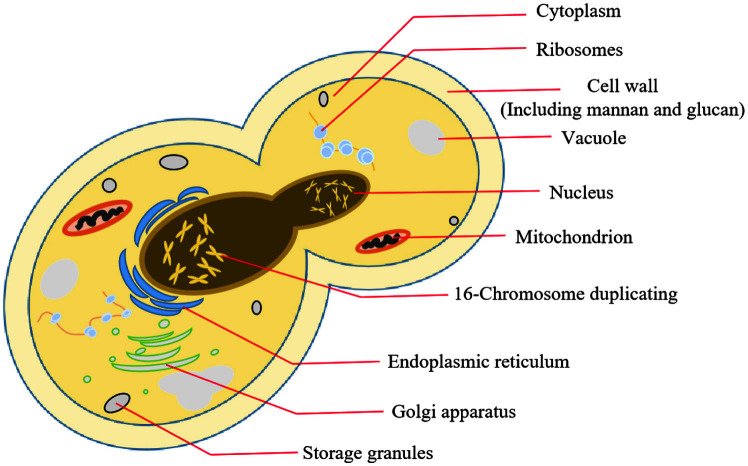
Schematic diagram of yeast structure.

**Fig. 2:**
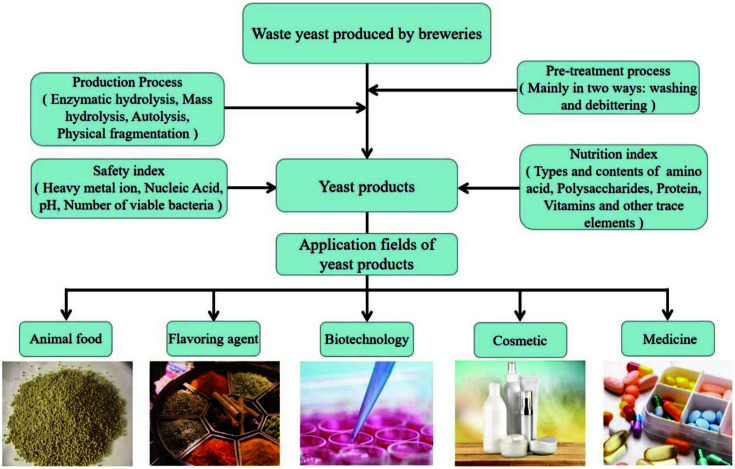
Conventional production process and application fields of yeast extract produced by breweries.

The type and characteristics of yeast extract depend on the waste yeast source it is made from and the particular production process used. For industrial production, various methods are used to disrupt the yeast cells, such as mechanical disruption, enzymatic lysis, organic solvents, or autolysis using salt as the solubilizer, and other autolysis methods, depending on the intended application [7]. The yeast raw materials used in the industrial production of yeast extract are mostly brewer's yeast and baker's yeast (Fig. 1), both of which come from completely different sources. Brewer's yeast is mainly obtained by fermenting waste yeast from breweries that produce beer, while baker's yeast requires special cultivation, is high in protein, with high safety and stability, so each has its own advantages and disadvantages. In terms of the frequency of use of these two yeasts, it is clear that brewer's yeast (*Saccharomyces cerevisiae*) is the most commonly used (Table 1) [8]. The brewer's yeast cell-wall contains a high proportion (15-30% of the dry cell mass) of cross-linked polysaccharides, mainly mannose oligosaccharides and β-glucan [9, 10]. The β-glucan product obtained from brewer’s yeast has antibacterial, antioxidant and various other biological activities, so not only is it used as a food additive and dietary supplement to promote digestion, it also enhances human immunity and reduces liver and blood lipids [11]. Similarly, baker’s yeast extract is rich in free amino acids, minerals and vitamins, and is also often used industrially to enhance the flavor of soups and sauces [12]. At present, waste brewer's yeast is the main one of many by-products in the brewing industry. On average, 150-200 tons of waste yeast pulp will be produced for every 10,000 tons of beer brewed [4]. For this reason, waste brewer's yeast has become the main raw material used in the production of yeast extract. Although yeast extract is low in fat and carbohydrates, rich in fiber, and widely used in animal-feed additives [6], its application in other fields is very limited. However, with improved production technology, the applications for yeast extract have expanded from animal feed into amino acid and protein products for human consumption, immunostimulants for fish farming, meat flavoring agents [11], spice additives, plant growth regulation, and soil conditioning (Fig. 2) [13].

Despite these broad application prospects of yeast extract, most related reviews are limited to its practical applications, which, however, do not necessarily fully exploit the unique characteristics of yeast extract, and there exists a disconnect between application and theory. In addition, there are very few reports on the pros and cons of the wide diversity of yeast extract production processes. In this review, the characteristics of yeast extract are summarized, the different production processes are compared and comprehensively reviewed, and recent research findings on yeast extract are also outlined and discussed.

## Composition and Characteristics of Yeast Extract

### Chemical Composition

Yeast extract is a very complex product, the main components of which are cell wall material and cell contents [6]. The cell wall is mainly composed of structural polysaccharides, such as mannose oligosaccharides and β-glucans, which are extensively cross-linked, and there are also small proportions of chitin and glycogen [10]. Most of these polysaccharides are water-insoluble and they make up as much as 83% of the total carbohydrate content of yeast cells [14]. The cell lysate contains a high proportion of essential and nonessential amino acids, ribonucleotides, minerals, vitamins, peptides, and other water-soluble substances (Table 2) [15]. The complexity of yeast extract is not only manifested in the different types of macro-molecules and small molecules it contains, but also in the diversity of the nutrient content. For example, for yeast extracts obtained from the same raw materials and production conditions, but with different processing times, there can be major differences in the product composition, as different production processes and raw materials result in even greater differences. In fact, it is these very differences in the production methods of yeast extracts that lead to the diversification of yeast extract products capable of meeting the needs of different industries and applications [16].

### Nutritional Characteristics

Yeast extract is high in nucleic acid, protein, B vitamin and fiber content [17]. As such, it is an important ingredient in animal feed as well as in dietary supplements to meet human nutritional requirements. Glucans, mannans, chitin, protein and other macromolecular substances derived from yeast extract provide more balanced nutritional supplementation to animal feed than plant-sourced supplements [18]. Moreover, ribose, the major reducing sugar in yeast extract, is an important precursor for cellular energy metabolism.

The addition of a suitable amount of yeast extract to poultry feed can strengthen the immunity of birds and reduce the incidence of disease [19, 20]. Many countries have banned the use in pig feed of spray-dried animal plasma (SDPP), which is a safety risk that is also expensive as a protein supplement [21], so many pig farmers have switched to yeast extracts that are safer and relatively inexpensive. Yeast extracts are also used in the daily feed of weaner piglets to meet their nutritional needs and enhance their immunity [22]. The addition of yeast β-glucan to human dietary supplements can lower cholesterol and liver fat levels, as well as promote the proliferation of beneficial intestinal microflora [17, 23]. Yeast β-glucan has useful functional properties that can enhance food products, such as fruit drinks, biscuits, yogurt, chocolate, and jelly [11]. Although yeast extracts are rich in beneficial nutrients and are widely used in various industries, there are restrictions on the use of high nucleic acid content ingredients. Yeast has a nucleic acid content of up to 15%, 10 times that of human tissues. Excessive nucleic acid intake increases uric acid levels and can lead to hyperuricemia and gout [24]; the United Nations Protein Advisory Group recommends limiting nucleic acid intake to 2 g per day in the adult diet [25]. One way to reduce nucleic acid intake is to remove purines from foods by using silver complexes, or cuprous salt precipitation [26].

### Antioxidant Properties of Polysaccharide Structures in Yeast Cell Walls

The polysaccharide components (mannan and β-glucan) in the yeast cell wall make a major contribution to the antioxidant properties of yeast extract, through their ability to scavenge hydroxyl free radicals and superoxide anions [27]. In particular, modification of β-glucan, by sulfation [28], or phosphorylation [29], can markedly change its physicochemical properties and biological activities (Table 3), thereby further improving its antioxidant capacity. Mannan also has excellent antioxidant properties in humans and has immunostimulatory, anti-aging, anti-tumor and other health-beneficial effects [30]. These two polysaccharides with antioxidant function are both extracted from yeast cells. Industrial production of β-glucan and mannan from yeast is an ideal choice due to the abundance of raw materials and the product having less pollution and high purity [31].

There are various methods for extracting the polysaccharide components from yeast cell walls, and the method can be selected and/or modified to meet particular application requirements. Common polysaccharide extraction methods are alkaline, enzyme, ultrasonic, and microwave extraction (Table 4) [32]. The extracted cell wall polysaccharides are often combined with other antioxidants, such as selenium, amino acids, vitamins and their derivatives, for use in skin-care products that can increase stratum corneum hydration and reduce skin roughness. This method of formulating yeast extract polysaccharides has become the mainstream direction of choice for the development of antioxidant skin-care products [31].

### Special Antioxidant Properties

The antioxidant properties of yeast extract are not limited to the polysaccharide components of yeast cell walls, as the cellular contents of yeast also have antioxidant functions under specific environmental conditions [33]. For example, when live yeast is subjected to oxidative stress, the cells can absorb phenolic compounds (such as syringic acid, ferulic acid, caffeic acid, chlorogenic acid, cinnamic acid, gallic acid and (±) catechin) from the environment [6], to enhance their antioxidant defenses, which can improve the antioxidant properties of yeast extract to some extent [34]. This approach has been used to optimize the production of glutathione (GSH) (an important antioxidant in yeast extracts) by yeast cells [35], potentially enabling mass-production of GSH and reducing the production cost of yeast extract for antioxidant purposes for the food and beverage industry [36].

Research on the antioxidant properties of yeast extracts has also been extended to the cosmetics industry; yeast extract is usually combined with other cosmetic ingredients to formulate sun protection, moisturizing and exfoliating products, which also protect the skin from oxidative stress [31]. For comparison, the antioxidant capacity of yeast extract is ten times that of blueberries [31].

### Organoleptic Properties

Organoleptic properties are another important property of yeast extract. In fact, the flavors of yeast extract as a condiment mainly include meat flavor and barbecue flavor, but inevitably, bitterness and yeast taste remain after processing, which is not acceptable to everyone [5].

### Aroma Properties of Yeast Extract as a Flavoring Agent

Yeast extract has become the fourth most important natural food-flavoring agent, after monosodium glutamate, nucleotides and hydrolyzed protein [37]. Treatment of yeast extract with the Maillard reaction (a complex series of reactions between heat-treated sugars and amino acids), enables production of a variety of flavors, such as umami, salty, meaty and other flavors, mainly derived from the amino acids and peptides in the lysate [5]. The chemical compounds responsible for some of the various flavors of yeast extracts have been identified, for example: meat flavor is derived from 2-methyl-3-furanmethanol, 2-methyl-3-methyldithiofuran and nitrogen-containing compounds such as pyrazine and furan; baking aroma from 2-furan-methyl-mercaptan and 4-hydroxy-2,5-dimethyl-3-furanone [38]; creamy flavor from 2,3-butanedione; nutty flavor from trimethylpyrazine; and chocolate flavor from 3-methylbutyraldehyde [39]. The aroma characteristics of 48 flavor compounds, including aldehydes, ketones, alcohols, furans, and pyrazines in yeast extracts have been reported [40].

### Sensory Properties of Nucleotides

Nucleotides in yeast extract are one of the three major flavoring substances in yeast extract, in addition to amino acids and peptides [24]. Although nucleotides do not have much flavor, they make a major contribution to the taste of yeast products by interacting with other components. Nucleotides based on 5'-adenosine phosphate (AMP), 5'-inosine phosphate (IMP), and 5'-guanosine phosphate (GMP) are 100 times more taste-active than seasonings such as monosodium glutamate [41, 42], so nucleotides play an important role in yeast extract food-flavoring agents.

### Flavor Modification Using the Maillard Reaction

Although yeast extracts made by different methods each have characteristic tastes and flavors, these properties appear to be closely related to the various nitrogen-containing compounds produced by the Maillard reaction [43]. The Maillard reaction is normally a by-product of cooking and heat treatment, but the resulting taste/flavor can be modified by changing the reaction conditions, such as the pH, salt concentration, the peptide concentration and composition, and the type of sugar (glucose, fructose, or sucrose) [44]. The intermediate products made from yeast extract using the Maillard reaction commonly include both volatile and non-volatile compounds. The non-volatile substances are usually amino acid derivatives, whereas the volatile substances include derivatives of alcohols, ethers, sulfur compounds, and aldehydes. The sulfur-containing volatiles generally make the greatest contribution to the overall flavor of most condiments [45]. Gas chromatography mass spectrometry (GC-MS) can be used to identify and characterize the key aroma-active substances produced by the Maillard reaction and the factors influencing their formation [5].

### Flavor Properties and Production of Glutathione

Along with the increasing application of the antioxidant and immune-stimulatory properties of glutathione (GSH), its properties as a flavor compound are becoming better known to the condiment industry. In recent years, industrial production of GSH using recombinant yeast cells obtained through genetic modification has become increasingly important [36]. As a precursor of a variety of flavor compounds, GSH is also gaining in importance for flavor modification over conventional yeast extracts [46].

### Improving Taste and Odor Defects of Yeast Extract

Although yeast extract is used as a food flavoring and seasoning, according to consumer surveys, there is an undesirable odor associated with it, which is repellant to some consumers and may limit sales of products containing yeast extract [40]. The challenge of odor removal from products made with yeast extract, such as nutritional supplements and condiments, is attracting increasing attention. Sensory evaluations of yeast extract have characterized its odor notes as burnt, sour, smoky, musty, gasoline and fatty [40], with most of these resulting from heat treatment at excessive temperature since the concentration of these odors increases with increased processing temperature [47]. The compounds mainly responsible for these odors are o-xylene, styrene, n-octanal and acetic acid; their relative concentrations vary depending on the yeast strain the extract was made from, treatment methods, and other factors [40].

The "yeast taste" in yeast extract is due to an important substance that affects its sensory evaluation and is related to one of its main odors, which is mainly composed of propionic acid and butyric acid. Ma *et al*. [48] used the mixed fermentation method of *Saccharomycopsis fibuligera* and *Lactococcus lactis* to completely remove propionic acid and butyric acid, the main sources of the "yeast taste." Under the action of these two bacteria, the lactic acid content with flavor and taste in the yeast extract increased by 6.27 g/l, so that the yeast extract showed improved flavor and taste, without having the "yeast taste."

In common with many protein hydrolysates, yeast extract has a bitter component to its taste and market surveys indicate that the bitterness is undesirable to most consumers [5]. The source of the bitterness is peptides resulting from hydrolysis of yeast proteins [49] and the intensity of the bitterness is generally proportional to the length of the peptide chain. Generally, heat treatment of foods can degrade long peptide chains, but heat treatment of yeast extract can strengthen the bitterness as the treatment temperature increases because the bitter peptides are very stable and heat- resistant [5]. However, limiting the heat treatment temperature to less than 120°C not only masks the bitterness but also strengthens the umami taste to produce a condiment with a much-improved taste [5]. Therefore, it is necessary for the food industry to strictly control all aspects of yeast extract production to meet food safety and flavor requirements. Future research on the sensory attributes of yeast extract should focus on further enhancing taste/flavor and eliminating odor and taste defects to maximize the market potential of yeast extract and make the most of its many positive characteristics.

## Yeast Extract Production Technology

**Fig. 3:**
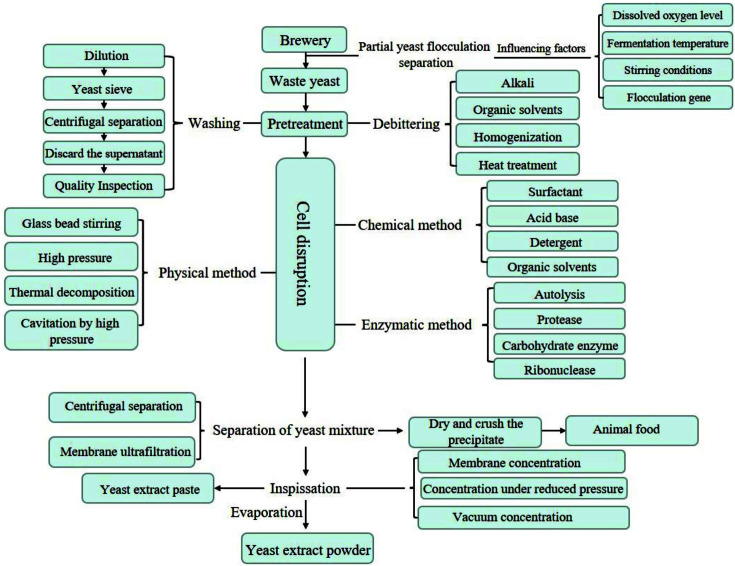
The production process of yeast extract with pretreatment, cell lysis, separation, inspissation, and evaporation.

Yeast cells have strong cell walls, so lysing the cells to release their contents is the main challenge in producing yeast extract. There are four main process types used to produce yeast extract (Fig. 3): autolysis, plasmolysis, enzymatic lysis, and physical methods [12], with each one having its own advantages and disadvantages (Table 5) [49, 50].

**Fig. 4:**
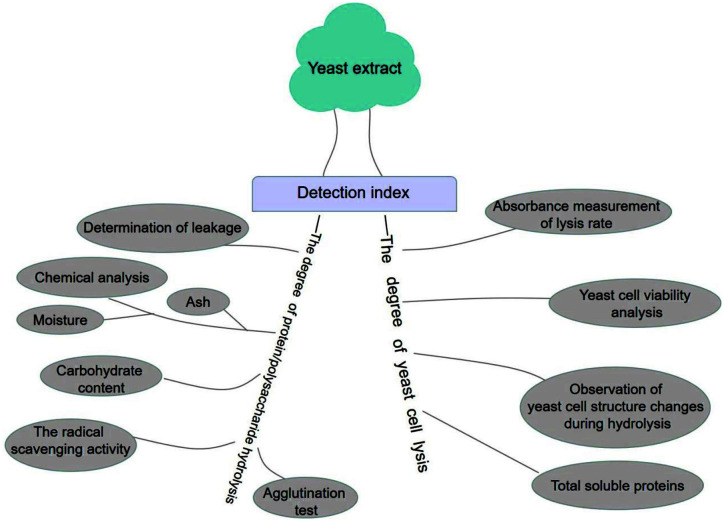
The detection index of yeast extract via the degree of yeast cell lysis and the degree of protein/polysaccharide hydrolysis.

Yeast extracts produced by different production processes from the same raw material can have marked differences in some of their properties, and therefore the choice of process must be carefully matched to the desired properties of the product. The standards commonly used to match processes and properties are measurements of the degree of yeast cell lysis (determined via cell morphology and cell viability assays) and the degree of protein/polysaccharide hydrolysis (determined via total soluble solid content, soluble protein content, and total carbohydrate content) (Fig. 4) [8].

### Pretreatment of Yeast for Extract Production

Under normal circumstances, pretreatment of waste yeast is required before large-scale industrial production of yeast extract, especially for food applications. Food-grade yeast extract has strict requirements for removal of toxic substances, or prevention of their formation, as well as removal of undesirable tastes and odors; therefore, pre-treatment is essential. The pretreatment methods generally involve washing and debittering. The first step is to wash the waste yeast to remove residues on the surface of yeast cells that may reduce product quality [51](mainly hop components, such as resin and tannin [52, 53] produced during the fermentation of beer by yeast)[54]. Spent yeast cells need to be washed multiple times with distilled water, diluted to 10% dry matter, filtered through a yeast sieve (mesh size 0.5 mm) and centrifuged to recover the cells, before undergoing quality and hygiene tests and the production process [4]. The second step is the debittering of the yeast cells. Most of the yeast used for extract production is waste yeast from beer fermentation. Beer yeast has a strong bitter taste due to the presence of humulones and isohumulones from the hops added to the fermentation to give the beer its characteristic bitter taste. These bitter compounds are mainly bound to the cell wall and therefore difficult to separate from the yeast by washing, especially at the low pH of the completed fermentation. The conventional method of debittering is to use high pH washing treatments or organic solvent/water mixtures [55]. Although the conventional method is simple to perform and low cost, it produces a large amount of toxic waste water.

A new yeast extraction process combines debittering with extraction, avoiding alkali-debittering, by taking advantage of the binding of bitter compounds to the cell wall at low pH [55] and using a combination of homogenization, autolysis and rotating microfiltration technology. This approach maximizes the release of cell contents, but the bitter components mostly remain attached to the cell-wall fragments, enabling high debittering efficiency along with a high yield of protein and other substances; this method has great application potential in industrial production.

### Yeast Extract Production via Autolysis

Autolysis involves endogenous yeast hydrolytic enzymes (mainly proteases, nucleases, and glucanases), which are activated by artificial methods and degrade the cell-wall polysaccharides, DNA, RNA, and cellular proteins [12]. During autolysis, there is a physiological change in which the activity of cellular respiratory enzymes decreases and that of the hydrolytic enzymes increases [56]. Common enzyme activators include ethanol, hexamethylenediamine, sodium chloride, Triton X-100 detergent, diethyl ether, digitonin, and sucrose. In the industrial context, although the use of organic solvents can increase product yield and permits efficient solid-liquid separation, there are also disadvantages, such as high cost and higher generation of polluting waste materials [12].

Autolysis usually involves suspending the yeast cells, addition of an activating agent, or heat treatment (40-60°C), stirring for 24 h at 50°C, centrifugal concentration, and recovery [7]. Although autolysis is much simpler than other cell lysis methods, it also has disadvantages, for example, low nutrient retention; autolysis is more destructive of antioxidant substances, *i.e.*, amino acids, B vitamins, polyphenols, and glutathione. However, if the yeast extract product application does not require a high content of nutrients, autolysis is the easiest, lowest-cost process for industrial production [57] and is the major production process for food-flavoring agents, which do not require a high nutrient content, but do need a high content of peptides and amino acids [11].

Autolysis has unique advantages over other yeast extraction processes for production of flavoring agents because the synergistic actions of a variety of proteases and peptidases increases the degree of protein hydrolysis and improves the amount and variety of free amino acids [58]. Autolysis not only produces better food-flavoring agents than other extraction methods, but careful control of the process conditions also allows the production of a much wider variety of flavors [59]. Among the many solubilizers, saponins are particularly typical [60]. They are natural emulsifiers that occur widely in leguminous plants. For example, in recent years, the use of quillaja (or “soapbark”, a tree native to Chile) saponins for efficient autolysis has been developed [61]. Saponin is a safe substance which has been officially approved for use in the food industry. Moreover, saponin can effectively avoid the disadvantage of promoting solvent in the yeast autolysis process. Taking salt- promoting autolysis as an example, in the production of yeast extract condiments or nutritional supplements, high salt content will seriously affect sensory characteristics and nutritional performance of the product. Using saponins to lyse yeast cells is effective, inexpensive, simple to implement, and provides a high yield of extract [61]. Saponins increase the permeability of the yeast cell membrane, allowing efficient release of cellular contents under mild conditions, and can promote protein degradation and the release of nitrogen-containing compounds at very low dosages. Compared with conventional autolysis, saponins can increase the release of cellular contents such as protein and the degradation of macromolecules by nearly 400 times [62]. A comparison of saponin/ethanol and saponin/sodium chloride mixtures to autolyze yeast cells shows that the addition of saponin markedly improves the preservation and release of nutrients from the cells [63] more so than the conventional high salt process [61].

In a radically different approach, the yeast cells are suspended in pure water and water absorption under the action of osmotic pressure causes the cells to rupture and release their contents; however, this method requires more development to improve cell lysis speed and nutrient preservation [64].

### Production via Plasmolysis

Plasmolysis involves the application of a cell membrane-disrupting agent (ethyl acetate, toluene, or ethanol) to the yeast cells to disrupt the integrity of the lipid bilayer and greatly increase the permeability of the cell membrane, permitting complete release of the cell contents into the external medium. Currently, the most common plasmolysis reagent is 1.5% ethyl acetate, the use of which is also referred to as an improved autolysis process and which works in a very similar manner to that of saponin-autolysis, as described above. In addition to promoting the autolysis of yeast, ethyl acetate has an inhibitory effect on the contamination of the yeast raw material [65]. Importantly, given the same raw material, the yield of extract obtained by plasmolysis with ethyl acetate is greater than that by the autolysis method [65].

The ethyl acetate plasmolysis method usually involves mixing of yeast cell suspension with ethyl acetate, adjustment of pH and temperature, further mixing for about 48 h, centrifugation, and finally analysis of the extract [8]. A comparison of the autolysis and plasmolysis methods, using the same spent yeast feedstock and analysis by cell viability, protein content, leakage analysis, and carbohydrate detection, found that plasmolysis produced a higher extract yield and higher solids content [8]. Similarly, a comparison of extract yield between plasmolysis with ethyl acetate/sodium chloride [58] and physical cell disruption by ultrasonic sonotrode found little difference in the total protein and residual ash contents [7]. This improved autolysis method (*i.e.*, plasmolysis) is significantly more efficient than the conventional autolysis process.

### Production via Enzymatic Degradation

Enzymatic degradation is very similar to autolysis, with both using mild conditions, and enzymes to lyse the cells. The difference is that enzymatic degradation uses exogenous enzymes, whereas autolysis uses endogenous yeast enzymes. The principle of enzymatic degradation is to allow the enzyme to digest the cell wall proteins, subjecting the cell to osmotic shock, or precipitating the cell wall protein to obtain the lysate [8]. The main types of enzymes used are protease, zymolyase, flavourzyme, helicase, pancreatin, and protamex. The most effective enzymes are fruit-sourced proteases, such as papain, ficin, and bromelain; however, since all proteases cleave peptide bonds with some degree of selectivity and produce mainly peptides, it is important to add just enough enzyme to complete the conversion to peptides. Once the enzyme has hydrolyzed all of the peptide bonds it is selective for, the reaction cannot proceed further, so additional enzyme has no effect on the hydrolysis and just increases the production costs [50]. Enzymatic hydrolysis with trypsin was compared with autolysis under the same conditions; trypsin had a synergistic effect with various cellular enzymes and greatly increased the rate of cell degradation. Similarly, in a comparison of autolysis, plasmolysis, and enzymatic degradation, enzymatic degradation released the most soluble substances and proteins from yeast cells. Enzymatic degradation also has advantages, such as rapid cell lysis, low salt content, and less product odor [8]. Industrially, enzymatic degradation often uses a mixture of several exogenous enzymes, resulting in faster cell lysis and macromolecule degradation, and a higher recovery of soluble substances. However, the use of such enzyme mixtures requires thorough optimization to maximize extraction efficiency and minimize enzyme consumption and costs [50].

### Production via Physical Disruption

The common methods of physical disruption of yeast cells include high-pressure homogenization, ultrasound, bead milling, and overweight method. The overweight method is new and derived from the osmotic shock crushing method. It mainly uses the osmotic pressure changes of different phases to exert pressure on the cells to cause breaking [4]. There are many types of equipment and methods available for industrial-scale physical disruption of yeast and all have strict requirements for their operating environment, but they are widely used, effective, relatively inexpensive to operate, and produce a high yield of nutrients [4]. Another advantage is avoidance of the damaging effects of organic solvents and salts on yeast cell components and nutrients, as well as minimal waste production. The polysaccharides β-glucan and mannan in the yeast cell wall have the beneficial biological activities of scavenging free radicals, delaying aging, and lowering blood cholesterol and lipid levels. Mechanical disruption is particularly effective for obtaining these products from yeast in good yield and high quality [6, 66].

Taking mechanical crushing with glass beads as an example, the crushing process roughly requires the following steps: first, the cell suspension is mixed with with glass beads of different specifications (1:2 mass ratio), then mixed in a vortex mixer at 4°C for 1 min and repeated 10 times, and finally centrifuged to obtain the precipitate and supernatant [67]. Mechanical disruption is superior to autolysis in a number of ways. The free long-chain fatty acid yield from physical cell disruption was higher than from autolysis, probably because of fatty acid degradation by the solvent used for autolysis [6]. In the industrial production of trehalose by yeast, physical cell disruption produces a higher yield of trehalose than autolysis [68]. Autolysis of yeast cells results in a much greater loss of vitamins, especially folic acid and antioxidants, such as phenolics and glutathione [69], compared with mechanical disruption, which appears to be the best choice for production of high nutrient/bioactive content extracts [7].

Mechanical disruption, however, does not promote proteolysis and the extracts it produces contain little peptide, or free amino acid, meaning that it is well-suited to producing extracts high in vitamins and antioxidants, but not for flavorings, which require a high content of amino acid [58].

### Other Factors Affecting Yeast Extract Production

Given that each of the yeast extraction methods discussed above has both advantages and disadvantages, a single method is often unable to produce an extract with the required composition and properties. Therefore, in industrial production, a combination of two or more methods may be applied to produce the desired product [70].

There can be significant variability in the yeast raw material depending on the supply source. For example, yeast extract produced using delayed yeast (a waste yeast raw material with longer fermentation and growth time than other waste yeasts) is significantly lower than waste yeast in ash and protein content [7], because of differences in fermentation time and consequent differences in the age and growth stage of the yeast. Therefore, selecting yeast of a suitable age and metabolic state can significantly improve the quality of the final product [71].

Yeast cells contain two main useful components; the cell wall, which is used in health products and cosmetics, and the small molecules and proteins in the cytoplasm. Relevant studies have shown that when extracting the polysaccharide component in the yeast cell wall, some extraction methods will cause the loss and destruction of some nutrients (such as vitamin B6, vitamin B9, and vitamin B12) in the yeast cell. On the contrary, the extraction of certain nutrients in yeast cells will also cause different degrees of damage to the polysaccharide structure of the yeast cell wall. So, to fully release the cell contents or maintain the physiological function and structure of polysaccharides, it is necessary to separate the cell wall and cytoplasm [6].

## Applications of Yeast Extract

**Fig. 5:**
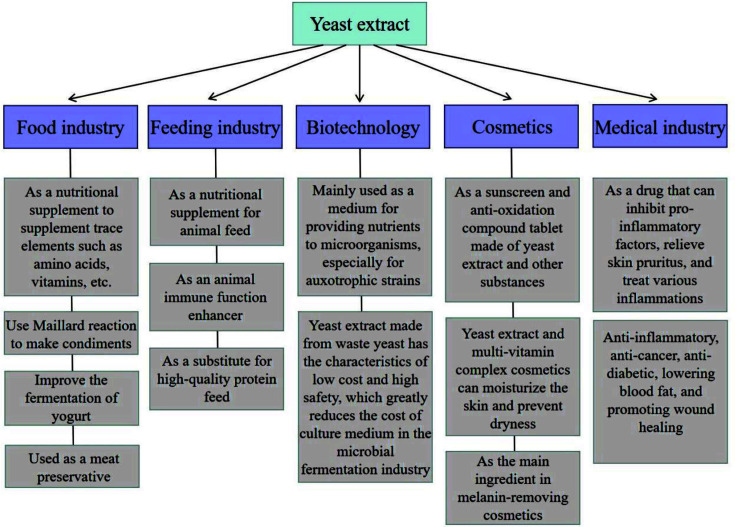
Characteristics of yeast extract and its application in various fields.

Yeast extracts have become increasingly prominent on the global market due to their unique nutritional and biochemical properties, low production costs, and abundant raw material supply from beer brewery wastes. They are widely used in animal feed, food, cosmetics, pharmaceuticals, health products, and biotechnology. Here, we summarize recent developments in application and the potential future research direction related to yeast extract (Fig. 5).

### Applications in Food

Yeast extract is rich in amino acids, peptides, vitamins, minerals, nucleotides, and other nutrients that are widely used as food-flavoring agents, food additives, and dietary nutritional supplements [11]. The essential amino acids in yeast extracts account for up to 40% of the total amino acids, which meets the UNFAO and WHO standards for the content of essential amino acids in healthy foods [72], and accounts for the extensive use of yeast extracts in nutritional foods [61].

Food-flavoring agents are an important application of yeast extracts in food, and the flavors of these agents are mainly meat flavor and barbecue flavor [73]. For example, variation in the reaction conditions for glutathione and its interaction with Maillard reactions produced a beef flavor and has been characterized as determining the main flavor components [46]. Heat treatment of yeast extract and optimization of the processing conditions produced a product with an umami/meat aroma; analysis by gas chromatography-olfactory-mass spectrometry found that furans and pyrazines were major contributors to the aroma [74]. Yeast extract-based seasonings were investigated as salt replacements, showing their potential to replace salt in foods while maintaining the original taste and nutritional value [40]. Yeast extract can also be useful in yogurt fermentation, which in combination with added yeast extract containing low-molecular-weight peptides promoted the growth of beneficial bacteria and shortened the fermentation time by 21% [75]. The addition of β-glucan from yeast extract to yogurt increased its thickness and improved its sensory evaluation scores [76].

In recent years, a new food additive has been developed, *i.e.*, yeast extract rich in phosphate (PO43-; polyPn, inorganic polyphosphate with an average chain length of n), which contains a high-energy phosphoric anhydride bond and is an effective meat preservative, which can keep fresh meat stable, soft, and juicy [77]. In particular, meat that has become tough can be restored to its original tender state by treatment with the high-phosphate extract [78].

### Applications in Animal Feed

Yeast extracts have long been recognized as a good source of nutrients in animal feed and are commonly used as a feed additive for poultry [79]. They can be produced to contain abundant polysaccharides, such as β-glucan, mannan [10], and chitin [80], which is ideal for poultry breeding and aquaculture, and act as a poultry immune-function enhancer [81, 82].

The livestock industry faces increasing problems, such as the need for ever faster growth, increasing animal morbidity, and the emergence of drug-resistant bacteria; some countries have banned the use of antibiotics and SDPP in animal feed. In response, there is increasing interest in immune-stimulating products, such as yeast extract, as substitutes for antibiotics and SDPP [83]. Yeast extract added to the daily feed of turkeys enhanced their growth and immunity by stimulating the oxidative burst activity of heterophilic cells and increasing red blood cell count, uric acid level, blood hemoglobin content, and other indicators [84]. Yeast extracts have great application potential for replacing antibiotic-based animal growth promoters [84, 85]. Yeast extract added to fish feed enhanced the immunity of Rosita fish species, increasing levels of white blood cells, serum proteins and globulins, and was more effective than brewer's yeast, or *spirulina* [83]. The active components were identified as β-glucan and mannan, which are antibacterial agents that regulate intestinal flora and stimulate immune system function, not only in animals, but also in humans [86, 87].

Yeast extract is also an important unconventional source of protein for animal feed [83]. Yeast extract and SDPP were added to pig feed as protein supplements for comparison of ileal digestibility, amino acid digestibility, metabolizable energy, and apparent digestibility: yeast extract was found superior to SDPP [88]. Yeast extract is likely to become a substitute protein ingredient in poultry feed in the future [22]. Yeast extract was combined with fish meal in different proportions as feed for farmed shrimp, as the proportion of yeast extract was increased, although no significant weight gain was observed, the digestive protease activity and the feed conversion rate of the shrimp increased, showing that yeast extract can replace up to 45% of fish meal and greatly reduce feed cost [89]. In summary, yeast extract is an immunomodulator and nutritional supplement product that has great potential to replace conventional protein ingredients in animal feed.

### Applications in Biotechnology

Yeast extract contains abundant amino acids, vitamins, nucleosides, polypeptides and minerals and is therefore an ideal nutrient growth medium for both laboratory and industrial microbial fermentation, especially for auxotrophic strains [90]. However, minor differences in the yeast raw material and the production process can result in major differences in the composition of the extract. For these reasons, careful selection of an extract suited to the organism of interest is very important.

Among the various nutrients in yeast extract, polypeptides are the key nutritional factors that affect the growth and metabolism of fungi, due to the fact that extracts can contain as many as 4,000 oligopeptides [91]. Even a small amount of peptide can markedly change the metabolic activity of bacteria during fermentation [92]. The peptides and amino acids in yeast extract are also main factors in the growth of lactic acid bacteria and can promote up to 60% of biomass [93, 94]. The auxotrophic *Streptococcus zooepidemicus* is the most commonly used strain for production of hyaluronic acid (HA), although the conventional fermentation medium is expensive and accounts for 80% of the production cost [95]. Therefore, finding a lower-cost fermentation medium is an important goal of the HA production industry [96]. Yeast extract not only meets the requirements of low production cost and ready availability, but also contains many key nutrients such as amino acids and vitamins needed for *S. zooepidemicus* fermentation, so yeast extract has become the standard growth medium for HA production [12]. Furthermore, due to the nutrient-rich properties of yeast extract, Hernández-Cortés *et al*. [97] found that by fermenting tequila with yeast extract instead of traditional agave juice, the trace elements and growth factors in the yeast extract relieved the nutritional limitation of agave juice on *S. cerevisiae* and the fermentation and aroma production capacity of *S. cerevisiae* was greatly improved.

A recent report stated that the stability of *Acidithiobacillus ferrooxidans* to the biological source of oil was significantly improved under the action of yeast extract as a culture additive. It reduces the leaching concentration of arsenic (As), a highly toxic waste in acid water, from 13.78 mg/l to 7.23 mg/l, and improves the stability by 40%, which is of great significance to the control of arsenic content [98].

Taking full advantage of the rich nutrients (amino acids, vitamins, carbohydrates) and safe and reliable properties of yeast extract, Shu *et al*. [99] innovatively applied yeast extract to nanoparticle technology to develop a new way to synthesize antibacterial silver nanoparticles (AgNPs). The AgNPs maintained their antibacterial ability while also being eco-friendly, safe, and non-toxic, which provides a new direction for the application of yeast extract.

As mentioned above, the composition and quality of yeast extract can vary depending on raw material and process differences. When two different yeast extracts were compared for culture of *Streptococcus thermophilus*, minor differences in extract composition resulted in differences in gene and protein expression, especially in the metabolic pathways of amino acid, citric acid, and urease [92]. Overall, yeast extract is inexpensive and often has an ideal nutrient composition for use by the fermentation industry; however, careful selection of a suitable extract is required for each micro-organism to optimize the fermentation.

### Applications in Cosmetics

Cosmetics are the basis of a huge and profitable industry, but they often have safety and efficacy problems, making it necessary to take great care and conduct rigorous testing when adding new ingredients to cosmetic products. Yeast extract is a well-established cosmetic ingredient; the amino acids, polysaccharides, polypeptides, proteins and other substances in yeast extract have beneficial biological effects, such as moisturizing the skin, promoting cell renewal, slowing skin aging, and speeding up wound healing, when applied topically [100, 101]. Yeast extract is usually combined with other substances, such as vitamins, moisturizers, and antioxidants, to achieve the desired cosmetic functions [102]. An oral tablet that combines vitamins and yeast extract has been developed to treat sunburn by reducing cell damage and lipid peroxide levels in the skin; yeast extract promotes cell renewal, so it has great potential for preventing photoaging and oxidative stress in the skin [103].

However, all new ingredients and products must be rigorously tested for safety and the absence of harmful side effects. For example, to test for potential skin irritation, yeast extract was combined with the common skin-care ingredients, including vitamins A, C, and E, and tested on the skin of healthy individuals. After several days of continuous use, there was no detectable skin irritation with any formulation and they all reduced skin roughness and increased the water content of the stratum corneum [31]. Although these preliminary findings are very positive, further testing will be required to confirm the safety of such formulations.

Yeast extract degrades and removes melanin from human skin and can be used in skin-lightening formulations. A common conventional skin lightener was compared with one containing natural active substances, such as yeast extract and salicylic acid. The two formulations were equally effective in reducing spot intensity and improving pigmentation, but the natural formulation has the advantages of extensive raw material resources and lower production costs. In general, the long-term tolerability of formulations containing natural ingredients like yeast extract is better than those containing synthetic chemicals, and the former have greater potential for future development [102]. It should be noted that the use of yeast extract in skin-care products is comparatively new and still restricted, and in addition, there are relatively few research reports on this area, so there is great potential for development of new applications and products.

### Applications in Medicine

Yeast extracts have found applications in the medical and healthcare fields, because of their biological activities, high nutritional content, activities in managing and preventing human diseases, and improving dysfunction of the intestinal microbial balance [104]. Commonly used anti-inflammatory and anti-bacterial treatments usually contain yeast extract [105], and β-glucan extracted from yeast cells has similar health benefits to the β-glucan from cereals. Yeast extract can be used to treat skin diseases, such as pruritus (itchy skin); about 13.5% of the world’s population suffers from this disease. A yeast extract formulation was compared with a conventional treatment, colloidal oatmeal lotion (CO), to treat pruritus, and the former was found to be very effective and superior to CO [106]. The efficacy of yeast extracts was mainly attributed to the flavonoids, dextran, amino acids, and vitamins it contains, which can block a variety of histamine receptors [107], thereby inhibiting pro-inflammatory factors, which alleviates itching [108].

The applications of yeast extract in medicine mostly relate to its anti-inflammatory properties, for example, in the treatment of emphysema and pneumonia [109]. In a mouse model of cigarette smoking, oral yeast extract significantly reduced numbers of the pro-inflammatory cells, neutrophils, eosinophils, and lymphocytes in the lung alveoli, as well as the content of the inflammatory mediators COX-2 and NOS, which was attributed to the antioxidant and anti-inflammatory properties of yeast extract [110]. There are still very few studies on the use of yeast extracts to alleviate pneumonia and other inflammatory lung conditions and further research is needed to explore its potential.

In addition to anti-inflammatory and anti-cancer effects, oral β-glucan has other health-beneficial functions, such as lowering cholesterol and blood lipid levels [111], without the side effects of synthetic drugs [112]. It also has an inhibitory effect on the formation and development of adipocytes, operating by inhibiting adipogenic differentiation [113]. In addition, obesity is closely related to the regulatory factors which control adipocyte differentiation [114, 115]. It appears that yeast β-glucan has great potential for development of treatments to manage conditions such as obesity, pneumonia, cardiovascular disease, and skin diseases.

## Conclusions and Perspectives

The development and utilization of yeast extract made from waste beer yeast has a history going back 70 years and large-scale yeast extract production is carried out around the world. Although the development of new, high-value applications for yeast extract is advancing, most of the production is still used in relatively low-value applications, such as animal feed and microbial culture. Application of yeast extract in nutritional supplements, medicine and cosmetics is still limited, and considerable further development is needed to maximize the high-value application potential of yeast extract.

Yeast extract is rich in nutrients, such as amino acids, vitamins and minerals, and is extensively used in food-flavoring agents and nutritional health products. The variety of extraction processes and conditions that can be used to produce yeast extract allows its composition to be tailored to specific applications by maximizing the content of nutrients, flavor compounds, bio-actives, or polysaccharides. There appears to be great potential for future process modifications to generate new flavor compounds and mixtures.

However, yeast extract has some disadvantages which limit its application potential. For example, the yeast raw material has a high content of nucleic acid and therefore a high content of purines. Excessive intake of purines increases the blood uric acid level, which increases the risk of gout and other health problems. Therefore, the technology for removing or reducing the level of nucleic acids in yeast extract still needs to be further developed and should be addressed by future research. Another problem with potential for improvement is the bitter taste of waste brewer’s yeast, caused by bitter compounds from the hops used in beer brewing adhering to the yeast cell wall.

As living standards in most countries have improved, consumer demand for healthy, nutritious and safe food has steadily increased, so future research should aim to maximize the great potential of yeast extract to meet these demands. One potential future research area is the use of metabolic engineering combined with multi-omics analysis methods to modify yeast metabolic pathways and optimize the intracellular composition of yeast; for example, by overproducing particularly valuable cellular components. However, this would not be possible using waste brewer’s yeast and new strains would have to be cultured specifically for extract production, thereby limiting this approach to particularly high-value applications.

## Figures and Tables

**Table 1:** Composition analysis of baker's yeast and brewer's yeast after treatment.

Yeast species	Treatment process	Condition	Yeast cell suspension solids Content (w/v%)	Components	Reference
Baker’s yeast	Autolysis	50°C, pH 5.0, 24 h	-	Protein: 52.5%, total solids: 1.98%	[116]
	Autolysis	55°C, pH 5.0, 2 h	13%	Total nitrogen: 11.2%, dry matter: 2%, β-glucan: 27%, trehalose: 1%	[117]
	Autolysis	55°C, pH 5.5, 48 h	15%	Protein: 14.4%, solids: 42.6%	[8]
	Autolysis and enzymatic hydrolysis	52°C, pH 5.2, 120 rpm for 72 h, then adding 2.5% papain and 0.025% lyase	50%	Protein: 56.75 g/l, solids: 59.84%, carbohydrate: 9.83 g/l	[50]
	Autolysis and enzymatic hydrolysis	57.5°C, pH 5.5, 2 h, then adding 0.6‰ papain and 0.2‰ β-glucanase	13%	Total nitrogen: 10.8%, dry matter: 2.25%, β-glucan: 27%, trehalose: 1.02%	[117]
	Plasmolysis	55°C, pH 5.5, 1.5% (v/v) ethyl acetate, 48 h	15%	Protein: 20.91%, solids: 45.2%	[8]
	Plasmolysis and enzymatic hydrolysis	48°C, pH 5.2, 1.5% ethyl acetate, 0.5% β-glucanase, 0.5% protease, 150 rpm for 24 h	18%	Solids: 51%, total nitrogen: 106 mg/g, α-amino nitrogen: 60 mg/g	[118]
	Enzymatic hydrolysis	55°C, pH 7.0, 0.2% (w/w) alkaline protease, 48 h	15%	Protein: 27.9%, solids: 52.1%	[8]
Brewer’s yeast	Autolysis	50°C, pH 6.0, 24 h	15%	Protein: 48.7%, solids: 56.8%, α-amino nitrogen: 3.9%	[61]
	Autolysis	55°C, pH 5.5, 50 h	11.25%	Protein: 32%, α-amino nitrogen: 4.9%	[119]
	Autolysis	50°C, pH 6.5, 20 h	18%	Total nitrogen: 8.2%, α-amino nitrogen: 4.5%	[120]
	Autolysis	70°C, pH 6.0, 4 h	-	Protein: 57.8%, sugar: 32.5%, ash: 6.9%	[121]
	Physical disruption	Glass bead breakage	-	Protein: 64%, solids: 14%, α-amino nitrogen: 3.79%, fat: 1.32%, carbohydrate: 12.9%, RNA: 4%	[6]
	Enzymatic hydrolysis	55°C, papain, 24 h	15%	Protein: 62.5%, sugar: 2.9%, fat: 0.1%, ash: 9.5%	[24]
	Enzymatic hydrolysis	10% phosphoric acid, pH 5.5. Firstly, adding 0.1% termamyl SC at 90°C for 1 h, then adding 0.1% SAN Super 240 at 55°C for 1 h, finally, adding 1.7% cellulase at 45°C for 10 h.	16.7%	Protein: 26.37%, fat: 8.18%, cellulose: 15.28%	[122]

**Table 2:** Types and contents of trace elements in yeast extract [6, 7, 121, 123].

Types of trace elements	Content (mg/100 g)
Alanine	3700-26600
Arginine	1680-12400
Aspartic acid	1370-11600
Cysteine	0-700
Glutamic acid	500-17500
Glycine	930-4900
Histidine	500-7300
Isoleucine	1750-5600
Leucine	3030-9000
Lysine	1660-9000
Methionine	500-2500
Phenylalanine	2640-5300
Proline	1850-4500
Serine	1360-6100
Threonine	200-6200
Tyrosine	400-5300
Valine	600-9100
Sodium (Na)	1.0-1356.3
Magnesium (Mg)	1.2-711.8
Calcium (Ca)	0.2-27.1
Potassium (K)	1.0-10000.0
Aluminium (Al)	0.1-1.1
Phosphorus (P)	0.5-3364.1
Nickel (Ni)	6.9-7.1
Strontium (Sr)	0.2-1.1
Lead (Pb)	8.7-9.7
Vanadium (V)	0.1-0.5
Selenium (Se)	0.03-23.92
Chromium (Cr)	0.010-0.019
Manganese (Mn)	0.6-15.9
Zinc (Zn)	4.6-22.6
Molybdenum (Mo)	0-0.002
Copper (Cu)	0.221-0.356
Cobalt (Co)	0.03-0.07
Silicon (Si)	83-118
Boron (B)	0.5-0.6
Thiamine (VB1)	0.0-20.0
Riboflavin (VB2)	0.0-2.4
Nicotinic acid (VB3)	68.2-597.9
Panthothenic acid (VB5)	4.4-20.3
Pyridoxine (VB6)	3.1-55.1
Biotin (VB7)	99.0-139.2
Folic acid (VB9)	1.4-5.0
Cobalamin (VB12)	0.1-0.3

**Table 3:** Comparison of different properties of β-glucan derivatives [28, 29]. ^[a]^

Types of β-glucan derivatives	Reduction capacity (700 nm)	Hydroxyl-radical scavenging rate	Anti-lipid peroxidation ability	Scavenging rate of superoxide anion
Sulfated β-glucan	0.3	38.45%	15%	35%
Phosphorylated β-glucan	0.05	67.59%	26%	65%
Sulfated-phosphorylated β-glucan	0.05	48.89%	7%	45%

^[a]^ The values in the table are all improved values over unmodified β-glucan.

**Table 4:** Different extraction methods for polysaccharides from yeast cell walls.

Extraction methods	Advantage	Disadvantage
Alkaline extraction	Short extraction time; low extraction cost; high product purity	The operation is cumbersome and requires strict control of the lye concentration and reaction time
Enzyme extraction	Simple operation; under the action of multiple enzymes, impurities such as chitin are completely removed, reducing the difficulty of subsequent separation	Multiple enzymes are required to work together and the enzymatic hydrolysis takes a long time (about 12 h)
Ultrasonic extraction	Low extraction temperature; short extraction time; convenient for subsequent product purification; no effect on the structure and physicochemical properties of the polysaccharides	The operation is complicated, and the extraction conditions need to be explored; when the temperature is too high, the properties of the polysaccharides will be destroyed; small processing capacity
Microwave extraction	High purity of extracted product; less waste is produced; mild reaction conditions	The operating conditions are strict, and the extraction temperature needs to be strictly controlled; the extraction cost is high; the processing volume is small, which is not suitable for mass production

**Table 5:** Comparison of different production methods of yeast extract.

Methods	Advantage	Disadvantage
Autolysis	Simple operation; low production cost; many types and contents of polypeptides and amino acids in the hydrolyzate; suitable for the production of flavoring agents	Low yield; difficulty in solid-liquid separation; poor taste as flavoring agent; microbial contamination; great damage to antioxidants; less nutrient retention
Plasmolysis	High solid recovery rate; strong antibacterial effect; reduced salt content in yeast extract powder; nutrients in yeast raw materials are completely released and preserved	Inefficient product conversion; solubilizers may impart off-flavors to products
Enzymatic degradation	Rapid degradation rate; more soluble substances after hydrolysis; high polypeptide content, low salt content and small odor	High hydrolysis cost; incomplete hydrolysis; required the coordination of multiple enzymes; long hydrolysis time; large damage to macromolecular substances such as proteins
Physical disruption	Simple operation; avoid the destruction of nutrients by organic solvents and salts; low byproducts; retain the activity of antioxidant substances	Required high operating environment; high energy consumption and high cost; low content of polypeptides and amino acids; not suitable for condiments
